# Effects of Treadmill Walking and Overground Walking in Young Children With Non-progressive Neurodevelopmental Disorders

**DOI:** 10.7759/cureus.71495

**Published:** 2024-10-14

**Authors:** Anvita Shiggavi, Komal KB, Navami Mahaveer

**Affiliations:** 1 Department of Physiotherapy, Bethel Medical Mission College, Bangalore, IND; 2 Department of Physiotherapy, KLE (Karnataka Lingayat Education) College of Physiotherapy, Hubli, IND

**Keywords:** cerebral palsy, conventional physiotherapy, gross motor functions, non-progressive neurodevelopmental disorders, overground walking, treadmill walking

## Abstract

Introduction

Neurodevelopmental disorders (NDDs) are responsible for childhood brain dysfunction and developmental disability. Physical therapists are important team members in providing a beneficial impact on the gross motor function of children with NDDs. Early intervention provides a beneficial effect on the improvement of gross motor function; however, most of the studies on treadmill training were in late age and the effect of early treadmill walking along with conventional physiotherapy was not studied. Hence, the objective of the present study was to compare the effect of overground walking with treadmill walking in young children aged 12-36 months with non-progressive NDDs.

Methodology

An experimental study was performed for one year on diagnosed cases of non-progressive NDD. A total of 36 children with non-progressive NDD were recruited for the study between the ages of 12 and 36 months of either gender and were allocated into treadmill walking and overground walking groups. The intervention was given for eight consecutive weeks, four days per week, and for 20-25 minutes per day. The children were assessed using Gross Motor Function Measure-66 (GMFM-66) and Peabody Developmental Motor Scale-2 (PDMS-2) for both stationary and locomotion components.

Results

The comparison of both the groups with PDMS-2 (stationary) at baseline, follow-up, and post intervention reported mean values of 25.56 ± 12.16, 27.22 ± 11.74, and 28.44 ± 11.72, respectively, in the overground walking group and 28.72 ± 8.96, 29.72 ± 9.46, and 31.33 ± 8.85 in the treadmill walking group, respectively, with statistically significant results (p = 0.0022). Additionally, the comparison of both the groups with PDMS-2 (locomotion) reported mean values of 35.78 ± 24.02, 39.11 ± 23.21, and 43.00 ± 23.58, respectively, in the overground walking group and 39.67 ± 22.28, 43.17 ± 22.94, and 46.61 ± 24.16 in the treadmill walking group, respectively, with statistically significant results (p = 0.020). Moreover, the comparison of both the groups with total GMFM-66 reported mean values of 16.97 ± 14.52, 18.35 ± 14.88, and 20.49 ± 16.86, respectively, in the overground walking group and 18.52 ± 14.28, 21.66 ± 15.76, and 25.92 ± 16.95 in the treadmill walking group with statistically significant results (p = 0.0118).

Conclusion

The present study concluded that between both the groups, a statistically significant difference was observed for PDMS-2 (stationary and locomotion) and GMFM-66 after a scheduled intervention of eight weeks. Hence, walking should be carried out as a part of therapy in early intervention and further studies can be performed to determine the long-term effect of these interventions and their role in the prevention of secondary complications.

## Introduction

Neurodevelopmental disorder (NDD) is defined as a genetic or acquired biological brain disorder or condition that is responsible for childhood-onset brain dysfunction. A developmental impairment such as autism, epilepsy, cerebral palsy (CP), or mental retardation may result from a severe NDD [1]. Based on their etiology, NDDs fall into three basic categories: acquired, genetic, and unknown [1]. An estimated prevalence of NDD among children aged two to nine years is 10% from hilly areas, 18% from rural, and 13% from urban areas [1]. Genetic causes include structural disorders consisting of tuberous sclerosis; chromosomal abnormalities including Down syndrome, Angelman syndrome, and Coffin-Lowry syndrome; and metabolic syndromes involving phenylketonuria. The acquired causes include arrested hydrocephalus, congenital rubella syndrome, traumatic injury, fetal distress syndrome, mechanical injury at birth, and postnatal infection [2]. NDD is classified into progressive and non-progressive NDD. In progressive NDD, the condition of the child deteriorates as the child grows and most commonly involves autosomal trisomies, Rett syndrome, mucopolysaccharidoses, and tuberous sclerosis. Whereas non-progressive NDD involves more commonly CP, Williams syndrome, Down syndrome, Angelman syndrome, West syndrome, cri du chat syndrome, and Prader-Willi syndrome [2].

The Gross Motor Function Classification System (GMFCS) evaluates the severity of motor involvement in CP, which comprises five levels depending on various initiated movements mainly in sitting, standing, and walking posture depending on the age group. A norm-referenced scale known as the Peabody Developmental Motor Scale-2 (PDMS-2) is used in early childhood to assess motor development [3]. Moreover, a criterion-referenced and functional-based scale known as the Gross Motor Function Measure-66 (GMFM-66) is used to assess the gross mobility of children with CP primarily and for documentation of various other NDDs between the age group of 0-18 years [4].

Delayed attainment in sitting, crawling, and walking is a common problem observed in NDD. The most commonly used physical therapy approach for children with locomotor disabilities caused by non-progressive NDD includes stretching, strengthening, weight-bearing exercises, passive standing on the standing frame, electrical stimulation, and mat exercises [5,6]. Task-specific training facilitates gross motor development as documented by neurophysiologic and early intervention literature [7].

Treadmill walking is an intervention that is used to promote the development or attainment of walking and also helps in the activation of existing spinal and supraspinal pattern generators in children with or at risk of non-progressive NDD [8]. Overground walking is a task-specific approach that helps to improve functional mobility, gross motor function, and functional performance [9]. Studies have been conducted to evaluate the effectiveness of partial body weight support treadmill training (PBWSTT) in children with CP along with comparative studies on treadmill training and overground walking among various age groups [9,10]. However, early intervention starting from the age group of 12 months to 36 months has not been performed in which an NDD group of children was considered [9]. Furthermore, there is a paucity of studies on the comparison of treadmill walking and overground walking on GMFM-66 and PDMS-2 (stationary and locomotion). Hence, the objective of the present study was to compare the effectiveness of treadmill training with overground walking in children of age group 12 months to 36 months with non-progressive NDD using evaluative scales GMFM-66 and PDMS-2.

## Materials and methods

After obtaining approval from the Institutional Ethical Committee with reference number HHEIEC/REV/392/2023, an experimental study was performed at the Paediatric Physiotherapy Department of Bethel Medical Mission Hospital, Bangalore for one year. The inclusion criteria consisted of diagnosed cases of non-progressive NDD who should have attained head control, have a chronological age of 12 to 36 months of either gender, and be medically stable. Children with lower extremity fixed deformities (equinovarus, equinovalgus, flexion contractures at the knee, windswept deformity, and hip subluxation), unstable cardiopulmonary conditions like arrhythmias, congenital heart diseases, cystic fibrosis, history of uncontrolled seizures, history of orthopedic surgeries in the past six months, injections in the past six months, weight more than 40 kg, and having pain, wound, or discomfort in the lower limbs were excluded [11]. Hence, a total of 36 children were included in the present study through simple random sampling and were divided into two groups. Group A received overground walking along with conventional physiotherapy whereas, group B received treadmill walking and conventional physiotherapy.

A brief explanation about the study was given to the parent/caretaker prior to the assessment, and written consent was taken for participation in the study from the parent of the child. Children were allocated into groups by the concealed envelope method by the principal investigator. Among 36 envelopes, 18 envelopes contained stickers with “Group A” and the other 18 had “Group B.” The parents were then asked to pick the envelope and according to the group mentioned in the sticker, the children were allocated to the intervention.

In group A, individualized conventional physical therapy was given for 20 minutes along with overground walking, which included weight-bearing exercises and passive standing on a standing frame. Neuromuscular electrical stimulation was given using sufficient intensity to elicit muscle contraction and strengthen the muscles of the lower limbs. Vestibular ball exercises improve postural reactions like sitting balance, quadruped, and supine-to-sit transitions [12]. In mat exercises in which weight bearing was initiated in different positions such as quadruped, kneeling, and half kneeling, the movement was supported, and transitional sitting and sit-to-stand transitions of the movement were aided [13]. An area of 100 m was marked in OPD where the children were made to walk overground for 20 to 25 minutes with the help of a walking assistive device or orthosis, and support at the pelvis was provided by the therapist. The children who did not have orthosis were made to walk barefoot. The assistance to initiate weight shifts and placement of the foot was given if required. The child was encouraged to walk for a longer duration and faster in each session by offering toys and the ball was kept in front and encouraged walking by asking them to kick the ball (auditory and visual input). The distance walked by the child was noted down in each session. The speed was calculated and noted down in each session. This intervention was scheduled continuously for four days per week for eight weeks.

A monitored treadmill (5555 Pro, Fitness World, Noida, India) with pause and safety buttons that could record the distance walked and time throughout a training session was used in group B. With increments of 0.1 kmph, the treadmill had the lowest belt speed of 0.8 kmph. During treadmill walking, partial weight support was provided by a suspension harness system. The system consisted of a harness that provided support around the trunk and the pelvis of the child. This harness was hung vertically from slings that were fastened to a horizontal bar that was situated just above the child's head and supported by a vertical frame. Two slings that were fastened at shoulder height supported the harness vertically. The system was framed over the treadmill to assist the child in such a way that the feet of the child were placed on the center of the treadmill belt. The child was instructed to stand as straight as possible, and their support to the body weight was gradually reduced until they started to flex at the knees or hips. The treadmill at the slowest speed of 0.8 kmph was then started and gradually increased by 0.1 kmph (as tolerated) until they could walk forward comfortably. The children who had orthosis were made to walk with orthosis, and the children who did not have any orthosis were made to walk with shoes on. Support was provided at the pelvis and assistance was given to shift the weight or in the placement of foot by the therapist wherever required. The child was encouraged to walk for a longer duration and faster in each session by offering toys and the ball was kept in front and encouraged walking by asking them to kick the ball (auditory and visual input). The distance and speed at which the child walked were noted down in each session. Treadmill walking was given for 20 to 25 minutes along with individualized conventional physical therapy for 20 minutes, as described above. This intervention was scheduled for four days continuously per week for eight weeks.

The outcome measures assessed were GMFM-66 and PDMS-2 (stationary and locomotion), which were evaluated at baseline, after four weeks, and after eight weeks. For the administration of GMFM-66, user manuals of GMFM-66 and GMFM-88 were used, which provide a complete description of every item, all illustrations of the activity, and scoring criteria. Maximum three trials for each item were performed and spontaneous performances of any item were acceptable. In some instances, if the child refused to attempt an item, it was tried at the end of the test or in another session. It was also marked as “not tested” [4].

The guide to the item administration, which includes a detailed description of each item, all activity illustrations, and scoring criteria, was used to administer the PDMS-2. The instructions were given up to three times. Sometimes an item was readministered after another item if the child lost interest in it before the third trial. To summarize, every task was completed by the children until they obtained a score of two or received three attempts [3].

Each subtest's entrance point was noted in the examiner record booklet, where the item was chosen depending on the age of the children. The child was considered to be at the basal level when they consistently scored two or three items in a row. The test was evaluated backward if the child scored 0 or 1 or any of the first three items given initiating from the entry point and continuing until the child scored two or three items consecutively. Once the basal was established, the ceiling level was represented, and increasingly more challenging items were administered until the ceiling was achieved. The child received a ceiling after failing three consecutive items with a score of zero [3].

Statistical analysis

The statistical software used to analyze the data was SPSS version 21.0 (IBM Corp., Armonk, NY). The mean and standard deviation for the baseline attributes were used to assess the descriptive statistics. The Kolmogorov-Smirnov test was performed to compare the data to a normal distribution to determine whether the data were normal [14].

Analysis of variance (ANOVA) test was used to compare the two groups on PDMS-2 (stationary and locomotion), GMFM-66, walking distance, and speed from baseline to follow-up, follow-up to post-intervention, and baseline to post-intervention between the groups. This test was used to compare the differences in PDMS-2 (stationary and locomotion), GMFM-66, walking distance, and speed between baseline to follow-up, follow-up to post-intervention, and baseline to post-intervention within the groups.

## Results

The mean age of the patients in group A and group B was 1.95 ± 0.78 and 2.43 ± 0.72 years, respectively. Additionally, the mean value of body mass index (BMI) was found to be 13.28 ± 1.38 kg/m² in group A whereas in group B, the mean value was 14.14 ± 0.87 kg/m². The gender-wise distribution of the patients demonstrated that in group A and group B, the maximum number of patients were males consisting of 15 (83.33%) and 14 (77.77%) patients, respectively, involving a total of 29 (80.5%) male patients in the study. Whereas, in group A, three (16.66%) were females, and in group B, four (22.22%) were female patients involving a total of seven (19.44%) females in the present study. The diagnosed cases enrolled for group A in the present study consisted of 12 (66.67%) CP patients, one (5.56%) global developmental delay (GDD) patient, and five (27.78%) genetic and chromosomal disorder patients. Whereas, for group B, the present study involved 12 (66.67%) CP patients, three (16.67%) GDD patients, and three (16.67%) genetic and chromosomal disorder patients.

Additionally, in group A, the maximum number of participants were barefoot consisting of 14 (17.78%) patients, followed by four (22.22%) with an ankle-foot orthosis. Whereas, in group B, 10 (55.56%) were wearing shoes, and eight (44.44%) were with an ankle-foot orthosis. The GMFCS level in both the groups described that in group A, four (22.22%) patients were in level II, six (33.33%) patients were in level III, three (16.67%) patients were in level IV, and five (27.78%) patients were in level V. Whereas, in group B, one (5.56%) patient was in level I, seven (38.89%) patients were in level II, two (11.11%) patients were in level III, six (33.33%) patients were in level IV, and two (11.11%) patients were in level V. The comparison of both the groups with PDMS-2 (stationary) is illustrated in Table 1.

**Table 1:** Comparison of both the groups with PDMS-2 stationary component. PDMS-2: Peabody Developmental Motor Scale-2.

Groups	Baseline (mean ± SD)	Follow-up (mean ± SD)	Post-intervention (mean ± SD)	F-value	p-value
Group A (overground)	25.56 ± 12.16	27.22 ± 11.74	28.44 ± 11.72	19.331	0.0022 (S)
Group B (treadmill)	28.72 ± 8.96	29.72 ± 9.46	31.33 ± 8.85		

The comparison of both the groups with PDMS-2 (locomotion) is demonstrated in Table 2.

**Table 2:** Comparison of both the groups with PDMS-2 locomotion component. PDMS-2: Peabody Developmental Motor Scale-2.

Groups	Baseline (mean ± SD)	Follow-up (mean ± SD)	Post-intervention (mean ± SD)	F-value	p-value
Group A (overground)	35.78 ± 24.02	39.11 ± 23.21	43.00 ± 23.58	8.3756	0.020 (S)
Group B (treadmill)	39.67 ± 22.28	43.17 ± 22.94	46.61 ± 24.16		

The comparison of both the groups with total GMFM-66 is demonstrated in Table 3.

**Table 3:** Comparison of both the groups with total GMFM-66. GMFM-66: Gross Motor Function Measure-66.

Groups	Baseline (mean ± SD)	Follow-up (mean ± SD)	Post-intervention (mean ± SD)	F-value	p-value
Group A (overground)	16.97 ± 14.52	18.35 ± 14.88	20.49 ± 16.86	10.516	0.0118 (S)
Group B (treadmill)	18.52 ± 14.28	21.66 ± 15.76	25.92 ± 16.95		

The comparison of both the groups with walking speed (kmph) scores at baseline, follow-up, and post intervention demonstrated that in group A, the mean values were found to be 0.86 ± 0.18, 0.86 ± 0.19, and 0.89 ± 0.20, respectively. Whereas, in group B, the mean values were found to be 0.89 ± 0.09, 0.91 ± 0.09, and 0.95 ± 0.10, respectively. Moreover, the comparison of both the groups with walking distance (km) scores at baseline, follow-up, and post intervention demonstrated that in group A, the mean values were found to be 0.30 ± 0.07, 0.31 ± 0.07, and 0.32 ± 0.07, respectively. Whereas, in group B, the mean values were found to be 0.33 ± 0.05, 0.34 ± 0.04, and 0.35 ± 0.04, respectively.

## Discussion

The present study reported that between both the groups, a statistically significant difference between PDMS-2 (stationary and locomotion) and GMFM-66 was observed. CP accounted for 66.67% of patients depicting the highest prevalence in comparison to the non-CP children. A previous study conducted on the incidence of CP showed 2-2.5 per 1000 live births [15] and GDD affects nearly 10% in India [16], which corresponded well with the prevalence of CP and GDD depicted. Additionally, in the present study, the male population of children was more, which was found to be similar to the studies conducted in the past [17,18]. However, the present study involves a heterogeneous sample, which includes non-CP. Moreover, the present study showed that the BMI in both groups was significant (p < 0.5). It is well known that the development trends of CP patients differ from those of the general population [19]. In comparison to their peers in the general community, patients with CP have lower average weight, muscular mass, fat storage, and linear growth [19], which is congruous with the findings of the current study.

GMFCS is developed and validated for the classification of CP but this was used as a classification system in non-CP children as well in this study. Many studies classified the non-CP children using GMFCS, which states the functional level of the children but not as an outcome measure. Those without CP show distinct motor growth curves on the GMFM from those with CP, indicating distinct developmental pathways [20].

Many other studies were performed to compare treadmill walking with overground walking in CP from which one study included ambulant children with GMFCS level I-III [10]. Another study examined the effect on children with GMFCS level I-IV to test the efficacy of overground and treadmill walking [21]. Moreover, a previous study included children with GMFCS levels II-III [9]; however, in the present study, children with GMFCS levels I to V were included.

Studies indicate that initiating early intervention for children who are developmentally delayed leads to positive outcomes in terms of growth across all developmental areas [22]. The fundamental rhythms of movement are generated by central pattern generators (CPGs), which are networks of spinal interneurons in the spinal cord that, in the absence of sensory input, produce perfectly timed sequences of α motor neuron activity. Every limb is equipped with a CPG array that functions as an oscillator. It has two half centers, one of which drives flexors and the other of which drives extensors in turn, with a reciprocal link between them. It was suggested in a prior study that children with CP have compromised supraspinal locomotor systems and CPGs. Walking on a treadmill stimulates these generators and processes of automatic reciprocation, a crucial component of gait that is performed through task-specific training [23]. Many studies supported the findings and illustrated that treadmill walking was effective [23-25]. Similarly, Begnoche et al. reported positive outcomes in enhancing the motor skills of children with CP from conventional physical therapy along with PBWSTT [26]. However, a study concluded that the PBWSTT is not effective in improving function and walking in children [27], which correlated with a previous study by Swe et al. [9]. In recent advances, telerehabilitation strategies were found to be effective in enhancing the effect of conventional physical therapy in post-stroke spasticity [28].

Hence, in the present study, improvement in PDMS-2 and GMFM-66 within both the groups could be the effect of early intervention and neural plasticity as the conventional therapy was given in both the groups at the early ages, which affects/develops the overall gross motor function of the children, which could have been the reason to show the effect rather than purely treadmill or overground training. Thus, in the clinical set-up, walking should be a part of conventional therapy, which should be initiated as early as one year of age for a robust positive outcome.

Strengths and limitations

The present study reported a statistically significant difference between both the groups after scheduled early intervention of eight weeks. However, the study demonstrated certain limitations, which included no sham therapy or control group, gait was not assessed using objective outcome measure, partial body weight support using a harness was not consistent in all the children as it was an individualized program, and the long-term effect was not evaluated.

## Conclusions

The present study concluded that between both the groups, a statistically significant difference was observed for PDMS-2 (stationary and locomotion) and GMFM-66 after a scheduled intervention of eight weeks. Hence, walking should be carried out as a part of therapy in early intervention and further studies can be performed to find out the long-term effect of the treatment and their role in the prevention of secondary complications. Additionally, comparative studies can be carried out between treadmill walking and overground walking without the involvement of conventional physiotherapy. Moreover, the involvement of a control group can be considered. Furthermore, studies can be conducted on a single entity of NDDs.
